# Characterizing pain in patients with Fabry disease: findings from a web-based cross-sectional survey in the US

**DOI:** 10.1186/s13023-025-03812-2

**Published:** 2025-06-16

**Authors:** Eric Wallace, Dawn Laney, Ibrahim Warsi, Connie Baldwin, Jack Johnson, Joseph Kupferman, Pronabesh DasMahapatra, Nicole Lyn

**Affiliations:** 1 Division of Nephrology, Department of Medicine, University of Alabama, Birmingham, AL USA; 2 Department of Human Genetics, School of Medicine, Emory University, Atlanta, GA USA; 3 Sanofi, Cambridge, MA USA; 4 Fabry Support & Information Group, Concordia, MO USA

**Keywords:** Abdominal pain, Fabry disease, Neuropathic pain, Pain, Pain crises, Patient-reported

## Abstract

**Background:**

Fabry disease (FD) is a rare, progressive disorder caused by pathogenic variants of the *GLA* gene resulting in the accumulation of toxic metabolites. Pain is a hallmark of FD, and patients often present with heterogeneous pain profiles. This cross-sectional, web-based survey was conducted to characterize pain and pain crises in patients with FD in the United States and explore the effects of sex, disease phenotypes, and treatment on pain.

**Results:**

A total of 66 participants (mean ± standard deviation [SD] age: 44.0 ± 12.7 years; females: 59.1%) completed the survey. Participants reported experiencing pain in upper (34.8%) and lower (43.9%) extremities several times a day and abdominal pain (31.8%) a few times a week. Overall, participants reported the nature of their pain as triggered (upper extremities: 47.0%; abdomen: 51.5%) or sudden (lower extremities: 57.6%). Female participants reported experiencing pain in upper (46.2%) and lower (48.7%) extremities several times a day and described it as sudden or triggered (48.7%) in upper extremities and sudden (61.5%) in lower extremities. Pain crises were reported in the lower extremities (80.0%), followed by the upper extremities (66.7%) and the abdomen (51.1%), and were often characterized as burning, tingling, or stabbing. A higher proportion of female participants (84.6%) than that of male participants (73.7%) reported pain crises in lower extremities. The duration of pain crises varied from 30 min to several days for different subgroups depending on sex and FD phenotypes. Most participants (81.0%) reported symptom improvement after 12 months of FD-specific treatment. Participants reported improvement in neuropathic symptoms (burning in hands, 45.9%), with an overall mean (± SD) satisfaction score of 7.2 (± 1.7) with agalsidase beta as the most recent medication.

**Conclusions:**

Pain was largely reported to be triggered across all subgroups. Consistent pain profiles were noted in participants across sex and FD phenotypes. Female participants reported pain burden similar to that of male participants, and pain crisis experience was heterogeneous across the subgroups. Most participants reported improvement in symptoms after FD-specific treatment and a high treatment satisfaction score with agalsidase beta.

**Supplementary Information:**

The online version contains supplementary material available at 10.1186/s13023-025-03812-2.

## Background

Fabry disease (FD) is a rare, progressive, X-linked genetic disorder caused by pathogenic variants of the *GLA* gene, leading to a deficiency in the lysosomal α-galactosidase-A enzyme [1]. This enzyme deficiency results in progressive accumulation of toxic metabolites, such as globotriaosylceramide and globotriaosylsphingosine [2, 3], and downstream activation of inflammatory pathways [4, 5]. A combination of these mechanisms leads to severe symptoms and life-threatening complications with an increased risk of mortality [6–8].

Symptom severity and onset in FD depends upon the pathogenic *GLA* variant and is typically classified into classic and non-classic/later-onset phenotypes (appearing in both male and female patients) [9]. The classic phenotype is characterized by early onset of symptoms with lower enzyme activity and involves multiple organs (noted in 1:22,000–1:40,000 male patients), while precise prevalence in female patients is unknown [6, 10–12]. Symptoms in the later-onset phenotype appear in adulthood and have less multi-organ involvement but can still be life-limiting [6, 10, 11]. Variants associated with this phenotype are observed in 1:1390–1:9372 individuals across different populations [13, 14].

In the United States, the primary treatments approved for managing patients with FD are intravenous enzyme replacement therapy and chaperone therapy. Enzyme replacement therapy includes agalsidase beta [15] or pegunigalsidase alfa [16]. One oral chaperone, migalastat, is available for patients with amenable variants of *GLA* [17]. Further, apart from these disease-specific treatments, adjunctive/symptomatic therapies, such as angiotensin-converting enzyme inhibitor, angiotensin receptor blocker, beta blocker, anticoagulant, pain management, and H2 blocker, are used to manage renal, cardiac, cerebrovascular, peripheral nervous system and gastrointestinal (GI) symptoms [6].

Clinical presentation of FD includes early symptoms of peripheral neuropathic pain (numbness, burning, tingling, and pain) [18] and GI symptoms (abdominal pain, bloating, nausea, constipation, and diarrhea) [19–21]. Pain is a characteristic of FD and is one of the early symptoms that can persist throughout life [22]. FD-related pain can be extremely distressing and is one of the significant determinants affecting patients’ quality of life [19] and disease burden [23]. Patients with FD experience both evoked and spontaneous pain, which can be chronic or episodic, indicating a range of pain presentation types [19, 22]. These manifest as varied pain symptoms, including, but not limited to, pain crises, hypersensitivity to mechanical stimuli, and neuropathic and GI pain, with high interindividual pain heterogeneity [19, 22, 24]. Patients often require analgesics as adjunctive therapies to manage their debilitating pain [25, 26].

Patients with FD also experience pain crises, described as intense, excruciating, and unbearable pain [26]. The most frequently described pain crisis begins in the lower or upper extremities and often radiates proximally [22, 27]. Although not traditionally considered a pain crisis, abdominal GI pain can also be acute-manifesting as severe abdominal pain triggered by meals or stress [20]. While pain crisis may not occur in all patients, it can be a critical diagnostic indicator of FD [26].

## Methods

### Study design

This observational, cross-sectional, web-based survey recruited patients with self-reported, physician-diagnosed FD in the United States. This study explored how patients with FD perceive their pain and pain crises, evaluated potential variations based on sex and phenotype, and aimed to understand the effect of current treatment on FD symptoms. The survey included male or female patients (≥ 18 years) experiencing moderate-to-severe neuropathic pain in the upper extremities, lower extremities, or abdomen, with the ability to read and understand English, and able/willing to provide electronic informed consent prior to participation. Patients who participated in another survey for an FD advocacy group in the past 2 months were excluded.

### Survey methodology

The survey was conducted among patients affiliated with the Fabry Support & Information Group, an FD patient advocacy group. An e-mail including the study invitation and a brief overview was shared with the eligible patients. Interested participants responded to the e-mail, and each received a unique weblink to administer the online survey. The survey was open for participation between April 8, 2021 and August 13, 2021. The estimated length of the survey was 30 min.

The survey received responses in four categories: participants’ eligibility, demographics and disease characteristics, pain characteristics (neuropathic and abdominal pain severity, frequency, duration, and nature), and FD symptoms and severity assessed using the Fabry Disease Patient-Reported Outcome (FD-PRO) questionnaire. The FD-PRO is a 19-item instrument that assesses symptom severity for adults with FD [28, 29]. Participants rated the severity of their symptoms in the past 24 h on a numeric rating scale from 0 (no symptom) to 10 (symptom at its worst). Participants’ satisfaction with the current treatment was also recorded on a scale from 0 (completely dissatisfied) to 10 (most satisfied). Participants could also select “don’t know/prefer not to answer” for any questions, as this survey was voluntary. For reporting purposes, these data have not been presented herein; however, only a maximum of 10% of participants chose this response for any question. The study received an institutional review board exemption under the 45 Code of Federal Regulations Part 46 from the Research Triangle Institute Office of Research Protection.

### Data analysis

All data collected *via* the survey were self-reported, and each question was analyzed individually among those participants who responded. Descriptive statistics were used to describe participants’ demographics, clinical characteristics, treatment history, FD treatment experience, pain profile, symptom severity, and bothersome symptoms. No imputation of missing data, statistical hypothesis testing, or sample weighting of questionnaire responses was carried out. Pain-related data were reported for the last 2 weeks prior to the survey completion date. Data were analyzed for the overall group and subgroups (based on sex and phenotype). Continuous variables were presented as mean and standard deviation (SD), and categorical variables as frequencies and percentages. All analyses were performed using SAS statistical software (version 9.4).

## Results

### Baseline characteristics

A total of 66 participants (mean age [± SD]: 44.0 [± 12.7] years; classic FD phenotype: 65.2%; females: 59.1%) completed the survey. The majority of participants with classic phenotype were female (*n* = 28; 65.1%) and 34.9% (*n* = 15) were male. The proportions of female and male patients in the later-onset phenotype groups were 47.1% (*n* = 8) and 52.9% (*n* = 9). The mean ± SD) time since diagnosis and time since diagnosis to the first treatment onset were 14.2 (± 11.2) years and 4.9 (± 7.4) years, respectively (Table 1).

**Table 1:** Self-reported baseline characteristics and clinical profile of participants with FD

Characteristics			Participants (*N* = 66)
**Age (years)**, mean (± SD)			44.0 (± 12.7)
**Sex**, ***n *(%)**			
Male			27 (40.9)
Female			39 (59.1)
**FD phenotype**, ***n *(%)**			
Classic			43 (65.2)
Male			15 (34.9)
Female			28 (65.1)
Later-onset			17 (25.8)
Male			9 (52.9)
Female			8 (47.1)
Not sure			6 (9.1)
Male			3 (50.0)
Female			3 (50.0)
**Time since diagnosis (years)**, mean (± SD) (*n* = 64)			14.2 (± 11.2)
**Time since diagnosis to first treatment (years)**, mean (± SD) (*n* = 56)			4.9 (± 7.4)
**eGFR (mL/min/1.73 m**^**2**^**)**, mean (± SD) (*n* = 26)			67.9 (± 33.0)
**CKD stage**^**a**^, ***n *(%)**			
Normal to mildly decreased kidney function			51 (77.3)
Moderate to very severely decreased kidney function			12 (18.2)
Not sure/Prefer not to answer			3 (4.5)
**Comorbidities**, ***n *(%)**			
Cardiovascular comorbidities^b^			32 (48.5)
No listed comorbidities			33 (50.0)
**Pain severity (PGIS)** ^**c**^	**Neuropathic pain**, ***n*(%)**		**Abdominal pain**, ***n*(%)**
Very severe	7 (10.6)		6 (9.1)
Severe	25 (37.9)		20 (30.3)
Moderate	26 (39.4)		23 (34.8)
Mild	8 (12.1)		12 (18.2)
No pain	0		5 (7.6)
**Pain medication used in the past 6 months**, ***n*(%)**			
Yes			56 (84.8)
No			10 (15.2)
**Type of pain medication used in the past 6 months**, ***n*(%)**			
Over-the-counter			32 (57.1)
Prescription only			34 (60.7)
Other			6 (10.7)
**FD-specific treatment status in the last 6 months**, ***n*(%)**			
Untreated (ever or in last 6 months)			9 (13.6)
Treated (currently or in last 6 months)			57 (86.4)
**Most recent FD treatment**, ***n*(%)**	**Agalsidase beta**	**Migalastat**	**Other**
**Overall (*n*** **= 58)**^**d**^	45 (77.6)	10 (17.2)	3 (5.2)

^a^CKD stage was self-reported and categorized as “normal-to-mild decreased kidney function” for Stages 1–2 and “moderate to very severely decreased kidney function” for Stages 3–5

^b^Prior myocardial infarction, prior stroke, transient ischemic attack, unstable angina, congestive heart failure, atrial fibrillation, coronary artery disease, arrhythmia, presence of a pacemaker, presence of a defibrillator, or other heart diseases

^c^Pain characterization for neuropathic pain in extremities and abdominal pain was assessed through a validated questionnaire [29]

^d^The most recent FD treatment can include patients who were treated more than 6 months ago (one patient in this case)

CKD, chronic kidney disease; eGFR, estimated glomerular filtration rate; FD, Fabry disease; *N*, total number of participants; *n*, number of participants; PGIS, Patient Global Impression of Severity; SD, standard deviation

Most participants (77.3%) reported normal-to-mildly decreased kidney function with a mean estimated glomerular filtration rate of 67.9 (± 33.0) mL/min. Participants reported their neuropathic and abdominal pain as mild (12.1% and 18.2%), moderate (39.4% and 34.8%), severe (37.9% and 30.3%), and very severe (10.6% and 9.1%). Most participants (86.4%) received FD treatment in the past 6 months or during the survey. Agalsidase beta was the most recent treatment reported by the majority of the participants (77.6%) (Table 1).

### Pain profile

#### Frequency and nature of pain profile

Overall, a high proportion of participants reported experiencing pain in the upper (34.8%) and lower (43.9%) extremities several times a day, whereas abdominal pain (31.8%) was mostly reported a few times a week (Table 2).

**Table 2:** Overall pain profile by frequency and nature based on location

Pain profile (*N* = 66)	Pain in upper extremities, *n* (%)	Pain in lower extremities, *n* (%)	Abdominal pain, *n* (%)
Pain frequency			
Several times a day	23 (34.8)	29 (43.9)	13 (19.7)
Once daily	8 (12.1)	10 (15.2)	10 (15.2)
A few times a week	16 (24.2)	14 (21.2)	21 (31.8)
Once a week	5 (7.6)	2 (3.0)	9 (13.6)
Once in the past 2 weeks	7 (10.6)	5 (7.6)	6 (9.1)
I did not experience such pain in the past 2 weeks	7 (10.6)	6 (9.1)	6 (9.1)
**Nature of pain**			
Constant, chronic pain	11 (16.7)	21 (31.8)	10 (15.2)
Sudden episodes of pain without a known trigger (e.g., pain crises)	30 (45.5)	38 (57.6)	24 (36.4)
Pain triggered by something else, such as diet, exercise, or change in temperature	31 (47.0)	23 (34.8)	34 (51.5)
Other types of pain	7 (10.6)	9 (13.6)	9 (13.6)

*N*, total number of participants; *n*, number of participants

In female and classic phenotype subgroups, pain frequency in the extremities and abdomen showed a trend similar to that of the overall study population. In contrast, more male participants reported experiencing pain in the extremities and abdomen a few times a week. The majority of later-onset participants experienced pain in the lower extremities once daily. In contrast, pain in the upper extremities and abdomen was experienced up to a few times a week. It is noteworthy that a higher proportion of female participants reported pain in the upper (46.2% vs. 18.5%) and lower (48.7% vs. 37.0%) extremities several times a day than male participants (Supplementary Fig. 1).

Among all participants, the nature of pain in the upper extremities (47.0%) and abdomen (51.5%) was described as triggered (caused by some trigger, such as diet, exercise, or change in temperature), while pain in the lower extremities (57.6%) was reported as sudden (without a known trigger) (Table 2). Participants (female vs. male) reported the nature of pain as either sudden or triggered in the upper extremities (48.7% vs. 40.7%, or 48.7% vs. 44.4%), sudden in the lower extremities (61.5% vs. 51.9%), and triggered in the abdomen (48.7% vs. 55.6%).

In the classic phenotype subgroup, pain was described as sudden in the upper and lower extremities and triggered in the abdomen (53.5% each), while in the later-onset phenotype subgroup, the majority of participants described their pain as sudden in the lower extremities (70.6%) and triggered in the upper extremities and abdomen (47.1% each) (Supplementary Fig. 2).

#### Pain crises by location, sex, and Fabry disease phenotypes

Of the participants who reported experiencing pain crises (*n* = 45), majority reported crises in the lower extremities (80.0%), followed by the upper extremities (66.7%) and abdomen (51.1%).

Pain crisis distribution was heterogenous based on sex. Male participants relative to female ones predominantly reported pain crises in the upper extremities (73.7% vs. 61.5%) and abdomen (68.4% vs. 38.5%), whereas a higher proportion of female participants (relative to male ones) reported experiencing pain crises in the lower extremities (84.6% vs. 73.7%).

**Fig. 1:**
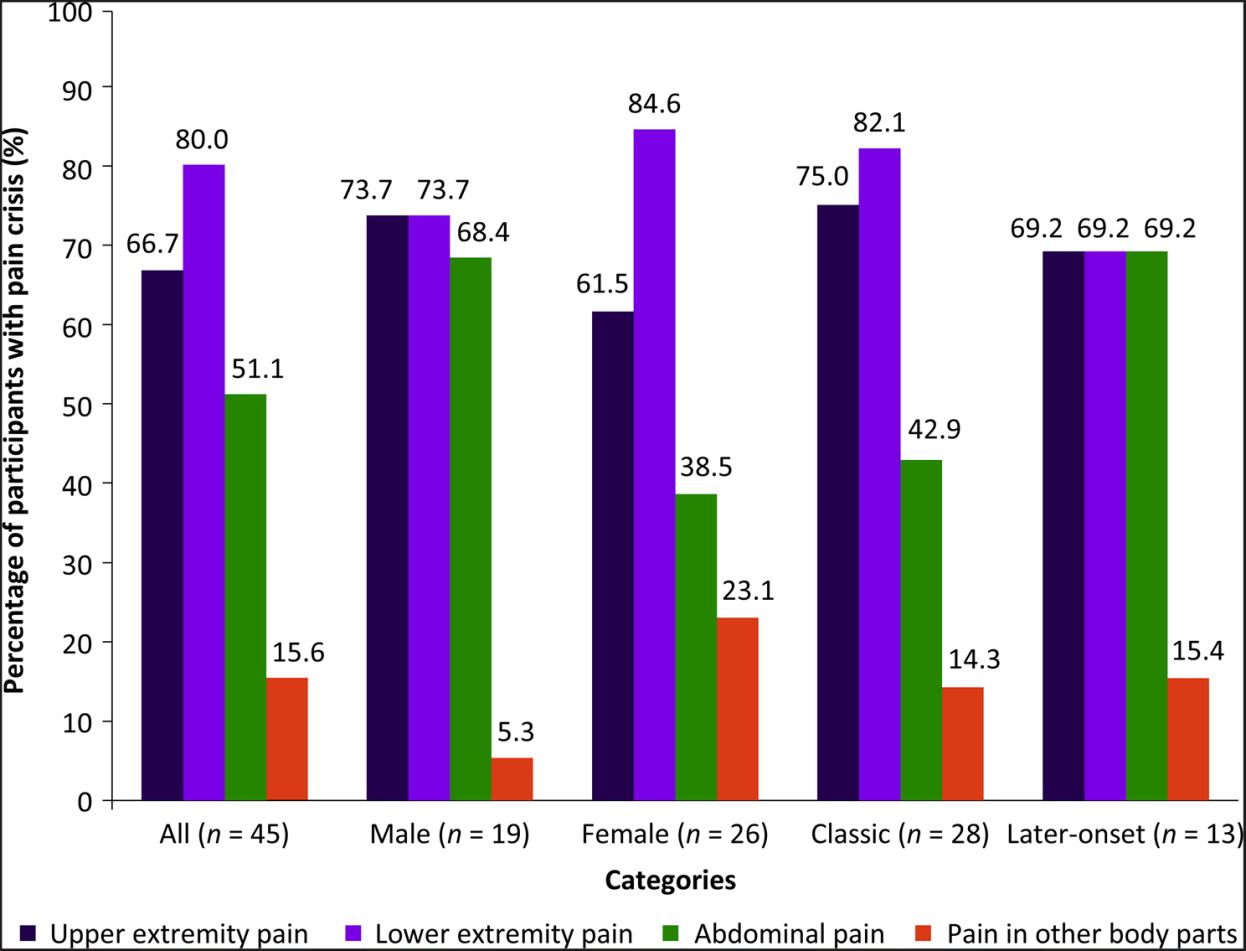
Pain crisis location by sex and phenotype in participants with FD. FD, Fabry disease; *n*, number of participants

The anatomical location of pain crises varied based on FD phenotypes. Participants from the classic phenotype subgroup reported experiencing pain crises mainly in the lower extremities (82.1%) and upper extremities (75.0%) compared to the abdomen (42.9%), whereas participants from the later-onset subgroup reported a similar proportion of pain crises (69.2%) across both the extremities and abdomen. Notably, abdominal pain crises were reported by a higher proportion of later-onset phenotype participants than classic phenotype participants (69.2% vs. 42.9%) (Fig. 1).

#### Pain crisis type and duration by location

##### Pain crises in extremities

Participants often reported pain crises in the lower and upper extremities as burning (30.3% each), tingling (27.3% and 24.2%), and stabbing (22.7% and 18.2%). The pain crisis duration varied depending on the anatomical location; it ranged from a few seconds to more than a week. Participants reported experiencing pain crises in the upper extremities for a few seconds (3.3%) to several days (20.0%) and in the lower extremities ranging from 30–60 min (25.0%) to a week (2.8%) (Table 3).

**Table 3:** Pain crisis type and duration profile by location

Pain crisis type	Pain crises in upper extremities, *n* (%)	Pain crises in lower extremities, *n* (%)	Pain crises in the abdomen, *n* (%)
Burning	20 (30.3)	20 (30.3)	1 (1.5)
Cold	3 (4.5)	7 (10.6)	5 (7.6)
Stabbing	12 (18.2)	15 (22.7)	12 (18.2)
Tingling	16 (24.2)	18 (27.3)	3 (4.5)
Shooting	13 (19.7)	17 (25.8)	3 (4.5)
Spreading	8 (12.1)	10 (15.2)	7 (10.6)
Prickling	10 (15.2)	8 (12.1)	0
None of these terms describes my pain crises	0	2 (3.0)	6 (9.1)
**Pain crisis duration**	**(*n*** **= 30)**	**(*n*** **= 36)**	**(*n*** **= 23)**
A few seconds	1 (3.3)	0	0
A few minutes	3 (10.0)	3 (8.3)	1 (4.3)
30–60 min	6 (20.0)	9 (25.0)	8 (34.8)
60 min to half a day	5 (16.7)	7 (19.4)	8 (34.8)
Between half a day and 1 day	4 (13.3)	8 (22.2)	2 (8.7)
Several days	6 (20.0)	6 (16.7)	3 (13.0)
A week	2 (6.7)	1 (2.8)	1 (4.3)
More than a week	3 (10.0)	2 (5.6)	0

*n*, number of participants

A consistent trend for the type and duration of pain crises was noted across sex and phenotypes. Most participants of both sexes characterized their pain crises as burning or tingling in the extremities. Participants with the classic phenotype often described their pain crises in the extremities as burning, while participants with the later-onset phenotype described their pain crises as shooting and spreading (Supplementary Fig. 3).

The majority of the male and later-onset subgroup participants reported experiencing pain crises in the extremities for half a day to 1 day and several days, while the majority of female and classic phenotype participants reported their pain crises lasting 30–60 min (Supplementary Fig. 4).

##### Pain crises in the abdomen

Pain crises in the abdomen were mainly characterized as stabbing (18.2%). They usually lasted 30–60 min (34.8%) or 60 min to half a day (34.8%) (Table 3). Across subgroups, pain crises in the abdomen were reported to have characterization and duration similar to those of the overall participants (Supplementary Fig. 3 and Fig. 4).

### Symptom severity and most bothersome symptoms

**Fig. 2:**
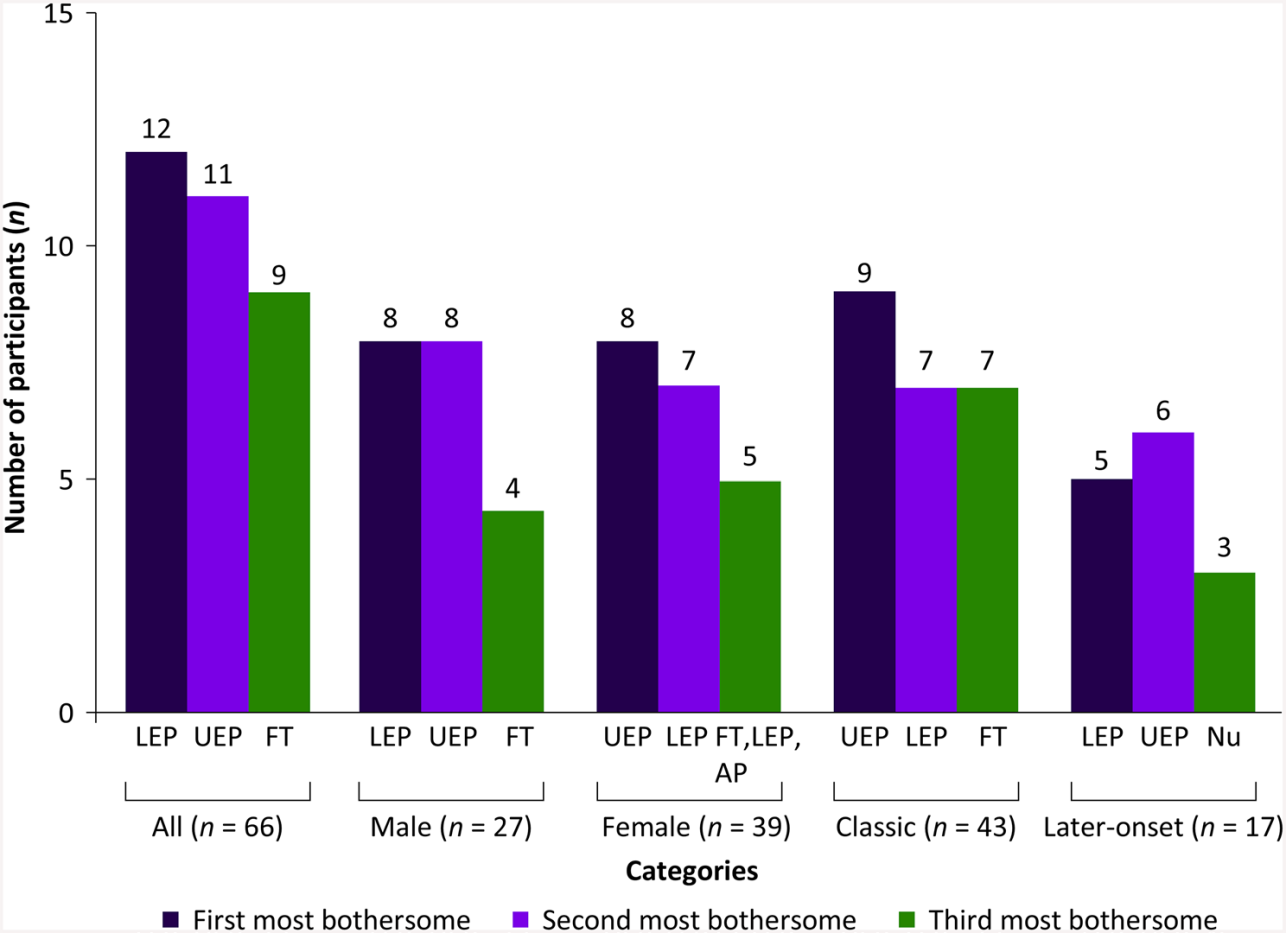
Most bothersome symptoms in participants with FD. AP, abdominal pain; FD, Fabry disease; FT, feeling tired; LEP, lower extremity pain; *n*, number of participants; Nu, numbness; UEP, upper extremity pain

Overall, the mean (± SD) FD-PRO symptom severity score was highest for the symptom of feeling tired [6.6 (± 2.3)], followed by pain in feet and legs [5.7 (± 2.7)] and feeling too hot [5.7 (± 2.8)] (Supplementary Table 1). Lower extremity pain was the most bothersome symptom across male and later-onset subgroup participants, while upper extremity pain was the most bothersome symptom for female and classic phenotype subgroup participants (Fig. 2).

### Symptom improvement with FD-specific treatment

#### Treatment improvement in participants over time

**Fig. 3:**
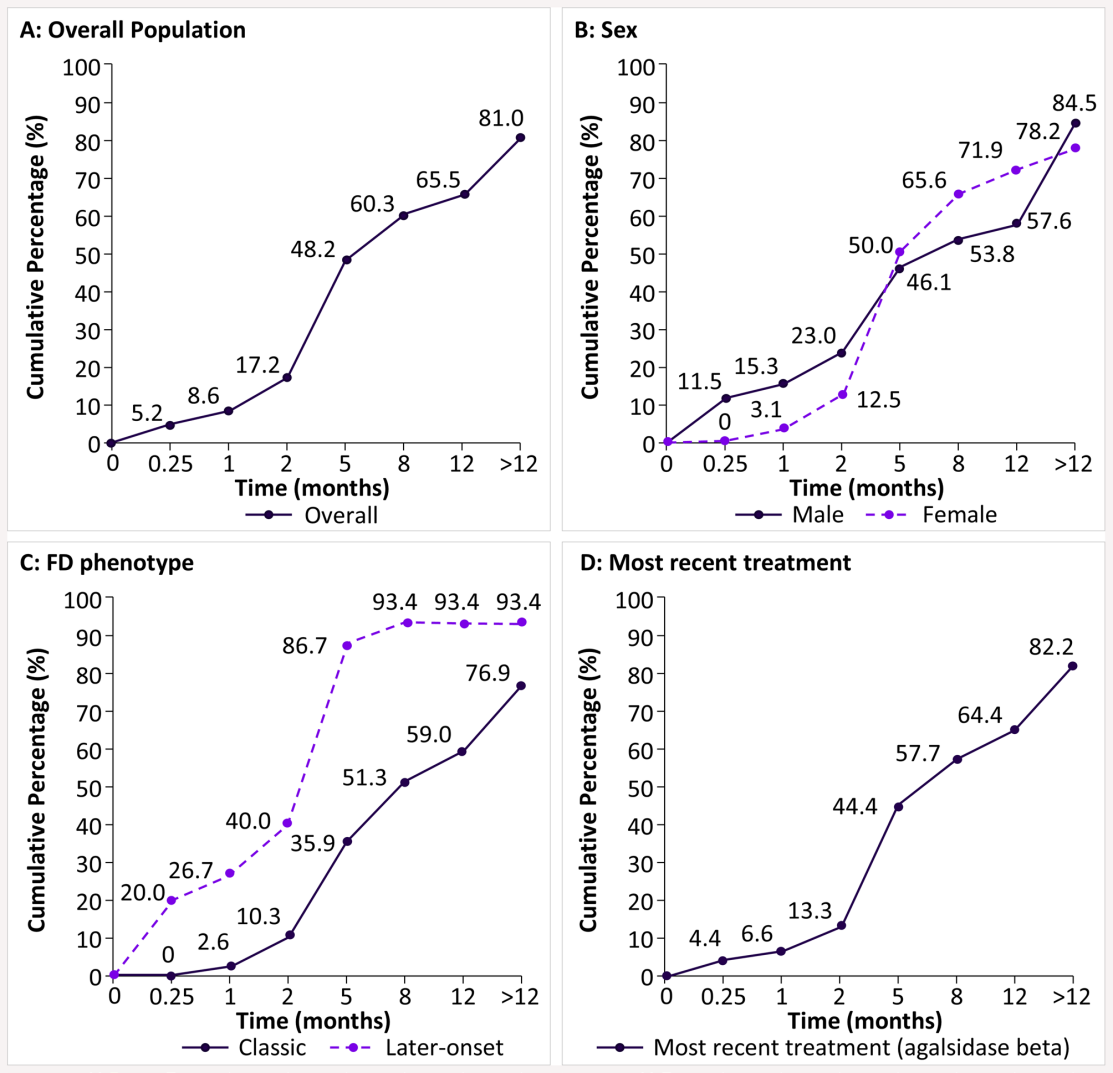
Cumulative time for treatment improvement in participants with FD. Note: Cumulative percentages were calculated by adding a percentage from one period to that of another period. Time until improvement was not presented to scale. The survey question was, “After initiating treatment with agalsidase beta, approximately how long did it take for you to notice an improvement in your symptoms?” Options provided for the participants were: Immediately; Less than a week; Between 1 week and less than a month; Between 1 month and less than 2 months; 2–5 months; 6–8 months; 9–11 months; 12 months; and more than a year. FD, Fabry disease

Overall, most of the participants (81.0%) with FD self-reported improvement in their symptoms after 12 months of FD-specific treatment (Fig. 3A). Cumulatively, after 5 months, 50.0% and 46.1% of participants from the male and female subgroups (Fig. 3B), respectively, and 86.7% and 35.9% of participants from the later-onset and classic phenotype subgroups (Fig. 3C), respectively, reported improvement. Of the participants who reported improvement with agalsidase beta, cumulatively, 4.4% reported improvement after 1 week of initiating treatment, 44.4% after 5 months, and 82.2% after 1 year (Fig. 3D).

#### Symptoms improved with agalsidase beta as the most recent treatment

**Fig. 4:**
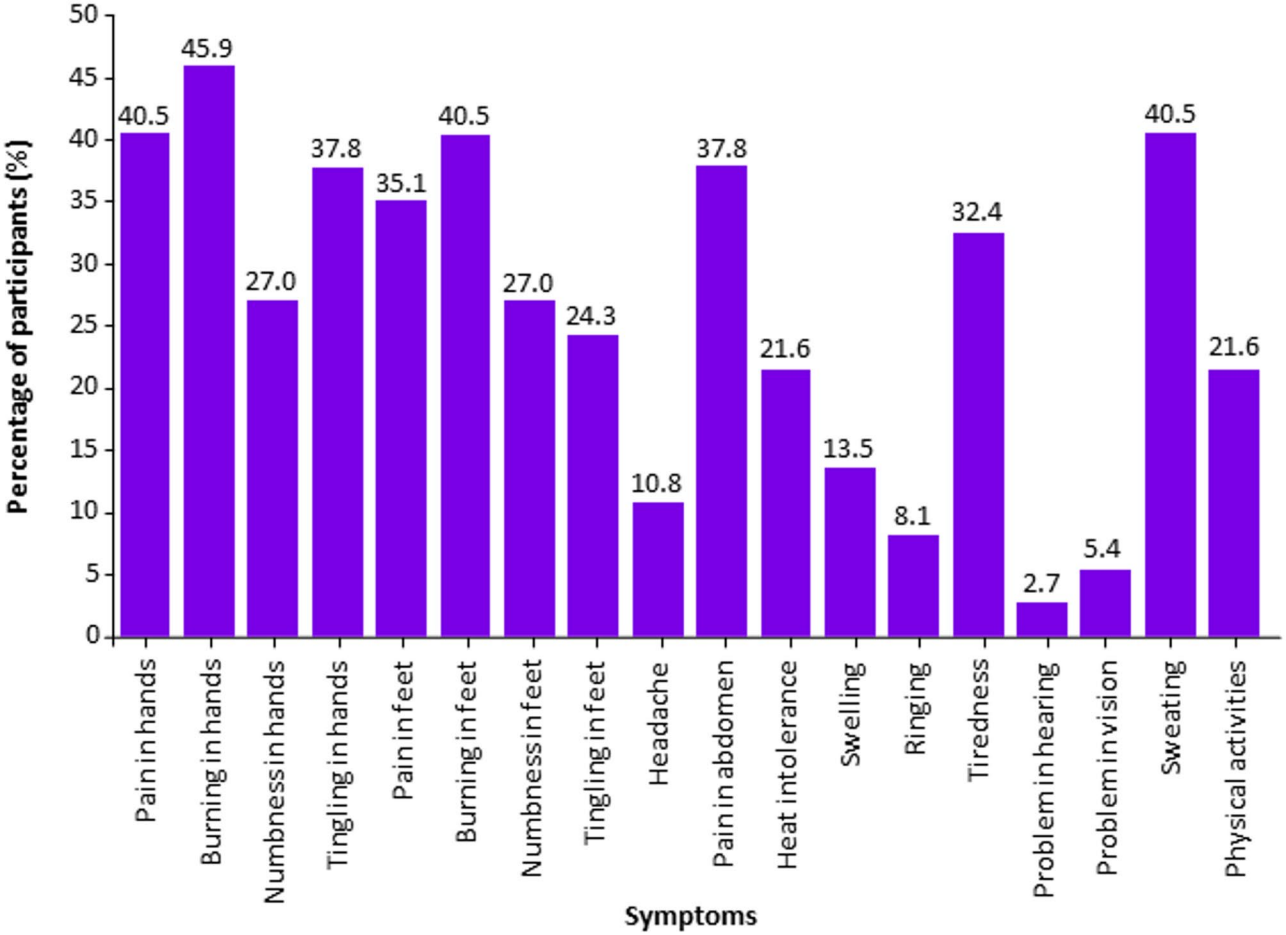
Participants with FD^a^ showing symptom improvement with agalsidase beta. ^a^Participants (*n* = 37) were allowed to select multiple symptoms. FD, Fabry disease

After receiving agalsidase beta, most participants reported improvement in their neuropathic symptoms, including burning in hands (45.9%); pain in hands, burning in feet, and sweating (40.5% each); and tingling in hands and pain in the abdomen (37.8% each) (Fig. 4).

#### Satisfaction with agalsidase beta

**Fig. 5:**
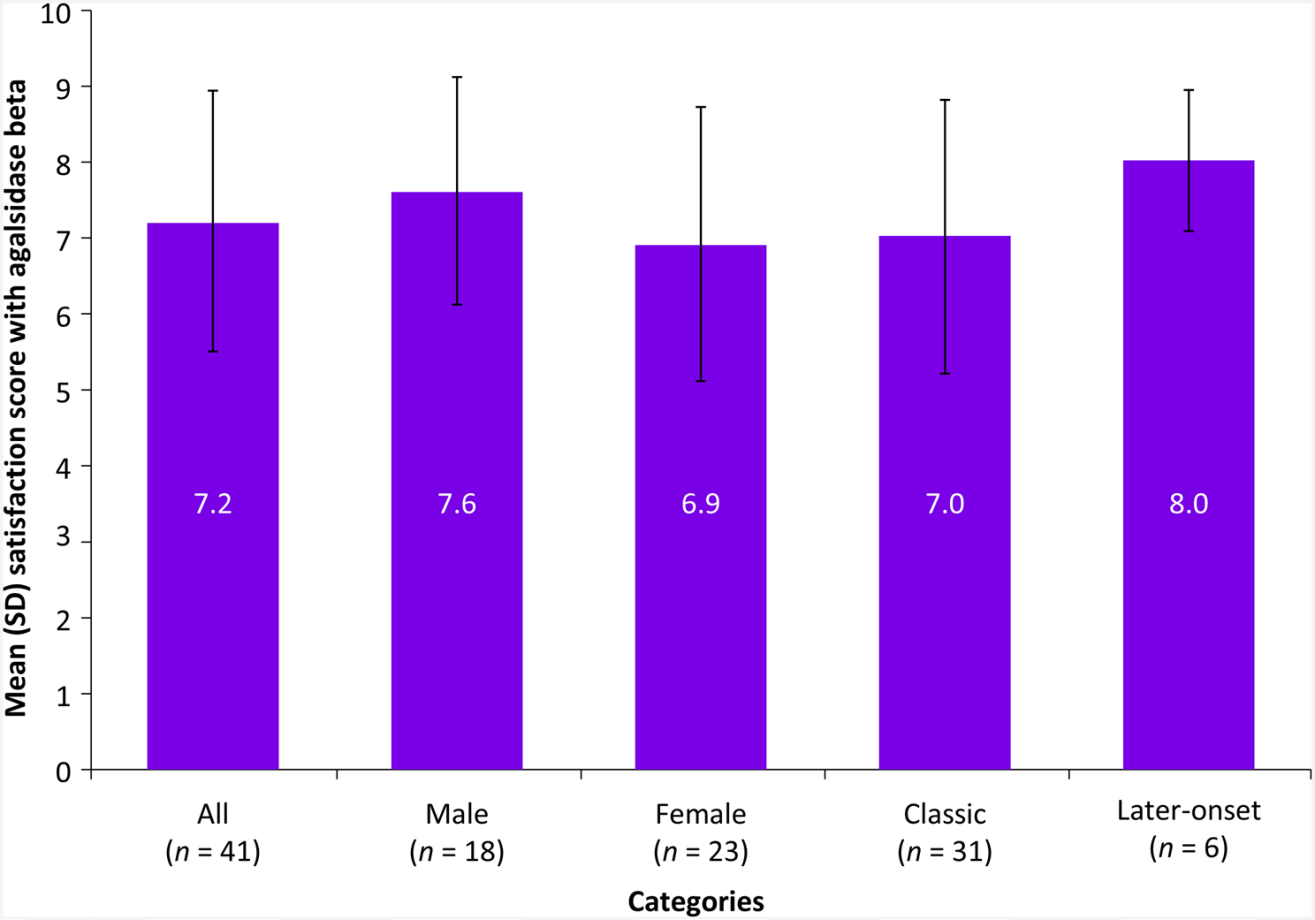
Satisfaction with agalsidase beta in participants with FD. FD, Fabry disease; n, number of participants; SD, standard deviation

The mean (± SD) satisfaction score was 7.2 (± 1.7) in participants who received agalsidase beta as the most recent treatment. Male [7.6 (± 1.5)] and later-onset [8.0 (± 0.9)] participants reported higher satisfaction than the overall population (Fig. 5).

## Discussion

Pain, whether neuropathic or functional, is one of the early symptoms of FD, and its occurrence may signal underlying organ deterioration [10, 20]. FD-related pain has been studied in the past in a variety of settings, including by using registries or screening of medical records [7, 22]. However, the present study analyzed pain and pain crises from a patient’s perspective with moderate-to-severe pain across different FD phenotypes and for both sexes. The study demonstrated a heavy burden of neuropathic pain, abdominal pain, and pain crises experienced across sex and phenotype subgroups.

Neuropathic pain is a hallmark of FD [26]; participants in this study reported frequent pain, especially in the distal extremities. Most participants characterized their pain as either triggered or sudden, which is similar to that in prior studies that had demonstrated a predominance of evoked neuropathic pain caused by stimuli that may not trigger pain in healthy individuals, indicating a high burden of pain associated with FD [23, 30].

Additionally, the present study demonstrated the pain crisis experience (nature, frequency, and duration) to be highly varied with regard to sex, phenotype, and anatomical location. The study indicated a heterogenous distribution of the duration of pain crisis, ranging from a few hours to a full day. Half of the participants experienced neuropathic crisis, predominantly in the lower extremities, and described it as burning in nature, while abdominal pain crisis was often described as stabbing, which also correlates with the results of a previous study reporting severe debilitating abdominal pain in patients with FD [20]. This novel characterization of crises in both sexes and for different phenotypes from a patient’s perspective furthers the understanding of the diverse experience of pain crises.

Prior data have often reported patients with later-onset FD phenotype to have less pronounced pain and sensory abnormalities when compared with the classic phenotype [9, 19, 31]. The present study, however, identified that among both phenotypes, the participants experienced neuropathic and abdominal pain; the participants from the later-onset phenotype even reported sudden pain in lower extremities at a higher proportion than the classic phenotype. Furthermore, contrary to published evidence that presents females experiencing mild and intermittent pain, the present study highlighted that female participants experienced severe pain in the upper and lower extremities as many as several times a day [30, 32, 33]. Thus, despite the previous and incorrect view of female patients with FD presenting with minimal symptoms [34], the present study showed that they may have a high pain burden and experienced pain symptoms similar to those of male patients. Thus, greater attention is warranted to address disease management for patients with later-onset FD and female patients with FD.

Pain in FD is associated with various mechanisms, although the pathophysiology of neuropathic pain in FD is not clearly understood. Small fiber neuropathy is reported to be the underlying cause of neuropathic pain and results from glycolipid accumulation in the dorsal root ganglia or the endothelial cells of the blood vessels supplying the nerve fibers [35–37]. While heterogenous experience of pain across sex, phenotype, and anatomical location in this study remains unclear, some studies have postulated the effect of sex hormones on pain intensity and the perception of pain [38, 39]. Increased male sex hormone, testosterone, has been associated with a decreased perception of pain and overall improvements in pain, whereas estrogen is linked with increasing hyperalgesia [38, 39]. Additionally, the early onset of the disease in male patients might lead to an early degeneration of nerve fiber terminals and can thereby result in attenuation of pain over time compared to that in the female patients. Moreover, the classical and later-onset phenotypes might be captured at different timepoints in male and female patients, thereby accounting for a different acuity stage of pain in these patients.

Pain in FD requires different therapeutic approaches through disease-specific treatment, supportive symptom management, and strategies to avoid pain triggers [26]. In the present study, most participants reported receiving pain medication (over the counter or prescription) in addition to FD-specific treatments for appropriate management of the multisystemic aspects of FD. Treatment guidelines recommend medications, such as carbamazepine, gabapentin, pregabalin, phenytoin, amitriptyline, duloxetine, and venlafaxine, and acute pain management with opioids (morphine, tramadol, and tilidate) [19, 22] for managing Fabry pain.

Further, considering at patient satisfaction with the current treatment, participants in this study mostly reported agalsidase beta as their most recent FD-specific treatment and were consistently satisfied with overall symptom improvement as expressed by 82.2% of participants by 1 year after the initiation of agalsidase beta. Previous studies also corroborated these findings, where agalsidase beta was beneficial in reducing pain and delaying the onset of renal, cardiovascular, and cerebrovascular events in patients with advanced FD [40, 41].

Pain experienced by patients with FD can disrupt their normal functions, including change in body weight, daily activities, and social interactions, thereby impacting their quality of life [22, 24]. The daily functions impacted by Fabry pain include eating, exercising, and learning [19, 42, 43]. Pain in patients with FD can result in moderate-to-severe impairment of mobility and ability to perform physical activities [36, 42]. In addition, Fabry pain can lead to high rates of fatigue and poor sleep quality [42]. There are reports of workplace absences by adults with FD or school absences by children with FD [28]. These impacts can lead to mental disturbances, social withdrawal, and poor quality of life. Morand et al. reported that an increase in pain intensity can progressively interfere with mood and general enjoyment of life [44]. Anxiety and depression are frequently associated with episodes of Fabry pain. There is an increased risk of depression in patients with FD due to neuropathic pain [45]. Thus, phenotypic heterogeneity of FD necessitates a multidisciplinary approach with a wide range of clinical specialties for effective pain management in FD [46].

There are certain inherent limitations of cross-sectional studies that we acknowledge, such as potential participant bias. Further, since all data in this study were self-reported, participants may have misspecified their FD phenotype. This study focused only on the descriptive analysis due to the small sample size; hence, the subgroup results do not imply any associations. The study enrolled patients who experienced moderate-to-severe neuropathic or abdominal pain, depicting a symptomatic population; therefore, the results are not generalizable to the overall FD population. The study could not characterize the impact of other treatments because of the small sample size. Nonetheless, this study provided a detailed analysis of pain and pain crises from a patient’s perspective, which may aid in better understanding and overall management of pain across FD patients.

## Conclusions

The present study showcases heterogenous pain presentation and, contrary to expectations, suggests that patients with later-onset FD, including female patients, experience pain and pain crises. Notably, female patients reported a pain burden similar to that of the male patients, adding to the increasing evidence that females with FD have high pain intensity. Further, the pain crisis experience of patients was heterogeneous among sex, phenotype, and anatomical location. Most participants on agalsidase beta reported symptom improvement and treatment satisfaction. Taken together, the present study highlights the importance of pain, a symptomatic manifestation of FD, which should be considered a critical indicator of FD across sex and phenotypes.

## Electronic supplementary material

Below is the link to the electronic supplementary material.

Supplementary Material 1

## Data Availability

The datasets used and/or analyzed during the current study are are included in this article and its supplementary information files. Patient level data will be anonymized and study documents will be redacted to protect the privacy of the participants.

## Abbreviations

AP: Abdominal pain
CKD: Chronic kidney disease
eGFR: Estimated glomerular filtration rate
FD: Fabry disease
FD-PRO: Fabry Disease Patient-Reported Outcome
FT: Feeling tired
GI: Gastrointestinal
GLA: Galactosidase alpha
LEP: Lower extremity pain
N: Total number of participants
n: Number of participants
Nu: Numbness
PGIS: Patient Global Impression of Severity
SD: Standard deviation
UEP: Upper extremity pain

## References

[1] Amodio F, Caiazza M, Monda E, Rubino M, Capodicasa L, Chiosi F, et al. An overview of molecular mechanisms in Fabry disease. Biomolecules. 2022;12(10):1460. 36291669 10.3390/biom12101460 PMC9599883

[2] El-Abassi R, Singhal D, England JD. Fabry’s disease. J Neurol Sci. 2014;344(1–2):5–19. 25106696 10.1016/j.jns.2014.06.029

[3] Najafian B, Tøndel C, Svarstad E, Gubler MC, Oliveira JP, Mauer M. Accumulation of globotriaosylceramide in podocytes in Fabry nephropathy is associated with progressive podocyte loss. J Am Soc Nephrol. 2020;31(4):865–75. 32127409 10.1681/ASN.2019050497 PMC7191924

[4] Rozenfeld P, Feriozzi S. Contribution of inflammatory pathways to Fabry disease pathogenesis. Mol Genet Metab. 2017;122(3):19–27. 28947349 10.1016/j.ymgme.2017.09.004

[5] Biancini GB, Vanzin CS, Rodrigues DB, Deon M, Ribas GS, Barschak AG, et al. Globotriaosylceramide is correlated with oxidative stress and inflammation in Fabry patients treated with enzyme replacement therapy. Biochim Biophys Acta. 2012;1822(2):226–32. 22085605 10.1016/j.bbadis.2011.11.001

[6] Ortiz A, Germain DP, Desnick RJ, Politei J, Mauer M, Burlina A, et al. Fabry disease revisited: management and treatment recommendations for adult patients. Mol Genet Metab. 2018;123(4):416–27. 29530533 10.1016/j.ymgme.2018.02.014

[7] MacDermot KD, Holmes A, Miners AH. Anderson-Fabry disease: clinical manifestations and impact of disease in a cohort of 60 obligate carrier females. J Med Genet. 2001;38(11):769–75. 11732485 10.1136/jmg.38.11.769 PMC1734754

[8] MacDermot KD, Holmes A, Miners AH. Anderson-Fabry disease: clinical manifestations and impact of disease in a cohort of 98 hemizygous males. J Med Genet. 2001;38(11):750–60. 11694547 10.1136/jmg.38.11.750 PMC1734761

[9] Arends M, Wanner C, Hughes D, Mehta A, Oder D, Watkinson OT, et al. Characterization of classical and nonclassical Fabry disease: A multicenter study. J Am Soc Nephrol. 2017;28(5):1631–41. 27979989 10.1681/ASN.2016090964 PMC5407735

[10] Germain DP. Fabry disease. Orphanet J Rare Dis. 2010;5(1):1–49. 21092187 10.1186/1750-1172-5-30 PMC3009617

[11] Azevedo O, Gago MF, Miltenberger-Miltenyi G, Robles AR, Costa MA, Pereira O, et al. Natural history of the late-onset phenotype of Fabry disease due to the p.F113L mutation. Mol Genet Metab Rep. 2020;22:100565. 32099817 10.1016/j.ymgmr.2020.100565 PMC7026617

[12] Bokhari SRA, Zulfiqar H, Hariz A. Fabry disease Treasure Island (FL): StatPearls Publishing; 2023 [Updated 2022 Dec 24]. https://www.ncbi.nlm.nih.gov/books/NBK435996/. Accessed Nov 12, 2023.

[13] Hwu WL, Chien YH, Lee NC, Chiang SC, Dobrovolny R, Huang AC, et al. Newborn screening for Fabry disease in Taiwan reveals a high incidence of the later-onset GLA mutation c.936 + 919G > A (IVS4 + 919G > A). Hum Mutat. 2009;30(10):1397–405. 19621417 10.1002/humu.21074 PMC2769558

[14] Wasserstein MP, Caggana M, Bailey SM, Desnick RJ, Edelmann L, Estrella L, et al. The new York pilot newborn screening program for lysosomal storage diseases: report of the first 65,000 infants. Genet Med. 2019;21(3):631–40. 30093709 10.1038/s41436-018-0129-y PMC6369014

[15] Prescribing information-Agalsidase beta. https://www.accessdata.fda.gov/drugsatfda_docs/label/2021/103979s5309lbl.pdf. Accessed Nov 12, 2023.

[16] Prescribing information- Pegunigalsidase alfa. https://www.accessdata.fda.gov/drugsatfda_docs/label/2023/761161s000lbl.pdf. Accessed Nov 12, 2023.

[17] Prescribing information-Migalastat. https://www.accessdata.fda.gov/drugsatfda_docs/label/2018/208623lbl.pdf. Accessed Nov 12, 2023.

[18] Burlina AP, Sims KB, Politei JM, Bennett GJ, Baron R, Sommer C, et al. Early diagnosis of peripheral nervous system involvement in Fabry disease and treatment of neuropathic pain: the report of an expert panel. BMC Neurol. 2011;11:61. 21619592 10.1186/1471-2377-11-61 PMC3126707

[19] Burand AJ Jr., Stucky CL. Fabry disease pain: patient and preclinical parallels. Pain. 2021;162(5):1305–21. 33259456 10.1097/j.pain.0000000000002152 PMC8054551

[20] Zar-Kessler C, Karaa A, Sims KB, Clarke V, Kuo B. Understanding the Gastrointestinal manifestations of Fabry disease: promoting prompt diagnosis. Th Adv Gastroenterol. 2016;9(4):626–34. 10.1177/1756283X16642936 PMC4913334 27366228

[21] Laney DA, Peck DS, Atherton AM, Manwaring LP, Christensen KM, Shankar SP, et al. Fabry disease in infancy and early childhood: a systematic literature review. Genet Med. 2015;17(5):323–30. 25232851 10.1038/gim.2014.120

[22] Üçeyler N, Ganendiran S, Kramer D, Sommer C. Characterization of pain in Fabry disease. Clin J Pain. 2014;30(10):915–20. 24121530 10.1097/AJP.0000000000000041

[23] Bashorum L, McCaughey G, Evans O, Humphries AC, Perry R, MacCulloch A. Burden associated with Fabry disease and its treatment in 12–15 year olds: results from a European survey. Orphanet J Rare Dis. 2022;17(1):266. 35840992 10.1186/s13023-022-02417-3 PMC9287883

[24] Rickert V, Kramer D, Schubert A-L, Sommer C, Wischmeyer E, Üçeyler N. Globotriaosylceramide-induced reduction of KCa1. 1 channel activity and activation of the Notch1 signaling pathway in skin fibroblasts of male Fabry patients with pain. Exp Neurol. 2020;324:113–34. 10.1016/j.expneurol.2019.113134 31778662

[25] Watt T, Burlina AP, Cazzorla C, Schönfeld D, Banikazemi M, Hopkin RJ, et al. Agalsidase beta treatment is associated with improved quality of life in patients with Fabry disease: findings from the Fabry registry. Genet Med. 2010;12(11):703–12. 20885332 10.1097/GIM.0b013e3181f13a4a

[26] Politei JM, Bouhassira D, Germain DP, Goizet C, Guerrero-Sola A, Hilz MJ, et al. Pain in Fabry disease: practical recommendations for diagnosis and treatment. CNS Neurosci Ther. 2016;22(7):568–76. 27297686 10.1111/cns.12542 PMC5071655

[27] Desnick RJ, Brady RO. Fabry disease in childhood. J Pediatr. 2004;144(5):S20–6. 15126980 10.1016/j.jpeds.2004.01.051

[28] Hamed A, DasMahapatra P, Lyn N, Gwaltney C, Hopkin RJ. Development of the Fabry disease Patient-Reported outcome (FD-PRO): a new instrument to measure the symptoms and impacts of Fabry disease. Orphanet J Rare Dis. 2021;16(1):285. 34172077 10.1186/s13023-021-01894-2 PMC8235809

[29] Hamed A, DasMahapatra P, Lyn N, Gwaltney C, Iaconangelo C, Serrano D, et al. Fabry disease Patient-Reported outcome (FD-PRO) demonstrates robust measurement properties for assessing symptom severity in Fabry disease. Mol Genet Metab Rep. 2021;29:100824. 34900595 10.1016/j.ymgmr.2021.100824 PMC8639795

[30] Gibas AL, Klatt R, Johnson J, Clarke JTR, Katz J. A survey of the pain experienced by males and females with Fabry disease. Pain Res Manag. 2006;11:828–964. 10.1155/2006/828964 PMC2539000 16960635

[31] Biegstraaten M, Hollak CE, Bakkers M, Faber CG, Aerts JM, van Schaik IN. Small fiber neuropathy in Fabry disease. Mol Genet Metab. 2012;106(2):135–41. 22497776 10.1016/j.ymgme.2012.03.010

[32] Chowdhury MM, Holt PJ. Pain in Anderson-Fabry’s disease. Lancet. 2001;357(9259):887. 11265986 10.1016/S0140-6736(05)71823-7

[33] Ro LS, Chen ST, Tang LM, Hsu WC, Chang HS, Huang CC. Current perception threshold testing in Fabry’s disease. Muscle Nerve. 1999;22(11):1531–7. 10514230 10.1002/(sici)1097-4598(199911)22:11<1531::aid-mus7>3.0.co;2-o

[34] Wang RY, Lelis A, Mirocha J, Wilcox WR. Heterozygous Fabry women are not just carriers, but have a significant burden of disease and impaired quality of life. Genet Med. 2007;9(1):34–45. 17224688 10.1097/gim.0b013e31802d8321

[35] Liguori R, Di Stasi V, Bugiardini E, Mignani R, Burlina A, Borsini W, et al. Small fiber neuropathy in female patients with Fabry disease. Muscle Nerve. 2010;41(3):409–12. 20120004 10.1002/mus.21606

[36] Torvin Møller A, Winther Bach F, Feldt-Rasmussen U, Rasmussen A, Hasholt L, Lan H, et al. Functional and structural nerve fiber findings in heterozygote patients with Fabry disease. Pain. 2009;145(1–2):237–45. 19665302 10.1016/j.pain.2009.06.032

[37] Schiffmann R. Neuropathy and Fabry disease: pathogenesis and enzyme replacement therapy. Acta Neurol Belg. 2006;106(2):61–5. 16898255

[38] Miclescu AA, Gkatziani P, Granlund P, Butler S, Gordh T. Sex-related differences in experimental pain sensitivity in subjects with painful or painless neuropathy after surgical repair of traumatic nerve injuries. Pain Rep. 2022;7(6):e1033. 36284797 10.1097/PR9.0000000000001033 PMC9586924

[39] Morgan J, Magwood K, Smith J, Jenkins MR, McGregor AJ, Quesnelle KM. Sex and gender differences in pain perception and management in clinical settings. All Life. 2024;17(1):2367421.

[40] Germain DP, Charrow J, Desnick RJ, Guffon N, Kempf J, Lachmann RH, et al. Ten-year outcome of enzyme replacement therapy with agalsidase beta in patients with Fabry disease. J Med Genet. 2015;52(5):353. 25795794 10.1136/jmedgenet-2014-102797 PMC4413801

[41] Banikazemi M, Bultas J, Waldek S, Wilcox WR, Whitley CB, McDonald M, et al. Agalsidase-beta therapy for advanced Fabry disease: a randomized trial. Ann Intern Med. 2007;146(2):77–86. 17179052 10.7326/0003-4819-146-2-200701160-00148

[42] Bouwman MG, Rombach SM, Schenk E, Sweeb A, Wijburg FA, Hollak CE, et al. Prevalence of symptoms in female Fabry disease patients: a case-control survey. J Inherit Metab Dis. 2012;35(5):891–8. 22431073 10.1007/s10545-011-9447-9 PMC3432199

[43] Hoffmann B, Schwarz M, Mehta A, Keshav S. Fabry outcome survey European investigators. Gastrointestinal symptoms in 342 patients with Fabry disease: prevalence and response to enzyme replacement therapy. Clin Gastroenterol Hepatol. 2007;5(12):1447–53. 17919989 10.1016/j.cgh.2007.08.012

[44] Morand O, Johnson J, Walter J, Atkinson L, Kline G, Frey A, et al. Symptoms and quality of life in patients with Fabry disease: results from an international patient survey. Adv Ther. 2019;36(10):2866–80. 31435831 10.1007/s12325-019-01061-x PMC6822826

[45] Körver S, Geurtsen GJ, Hollak CEM, van Schaik IN, Longo MGF, Lima MR, et al. Depressive symptoms in Fabry disease: the importance of coping, subjective health perception and pain. Orphanet J Rare Dis. 2020;15(1):28. 31992347 10.1186/s13023-020-1307-y PMC6986064

[46] Stepien KM, Broomfield A, Cole D, Deegan PB, Forshaw-Hulme S, Hughes D, et al. Management of pain in Fabry disease in the UK clinical setting: consensus findings from an expert Delphi panel. Orphanet J Rare Dis. 2023;18(1):203. 37480023 10.1186/s13023-023-02796-1 PMC10362568

