# Supplementary material for: Characterizing pain in patients with Fabry disease: findings from a web-based cross-sectional survey in the US
Source: Orphanet J Rare Dis. 2025 Jun 16;20:308. doi: 10.1186/s13023-025-03812-2 (PMC12172334; doi:10.1186/s13023-025-03812-2)
Supplement: Supplementary file 1 — Supplementary Material 1 [file 13023_2025_3812_MOESM1_ESM.docx]

# **Supplementary Material**


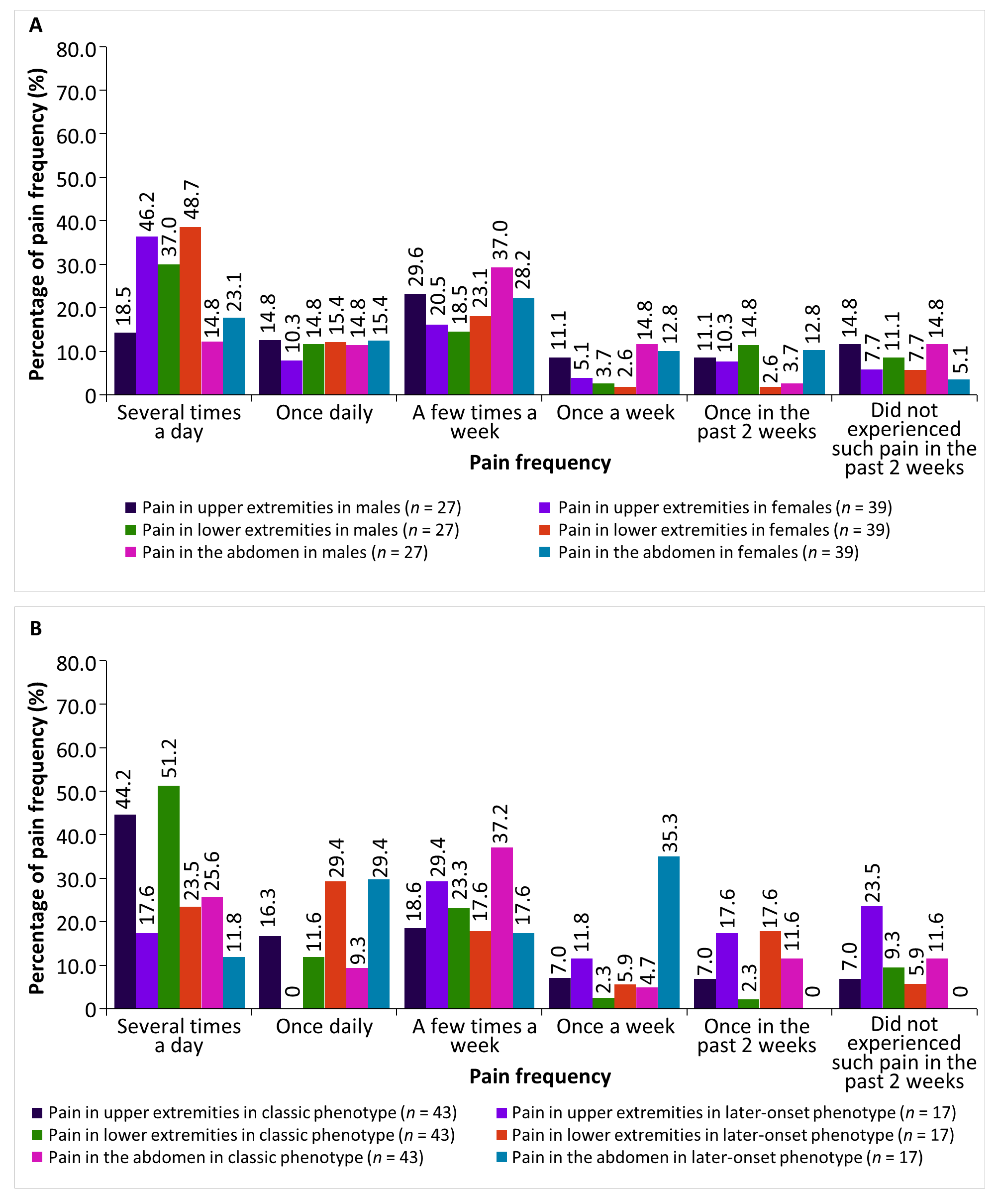


Supplementary Fig. 1 Pain frequency profile across sex (A) and phenotype (B) by location in participants with FD. FD, Fabry disease; *n*, number of participants


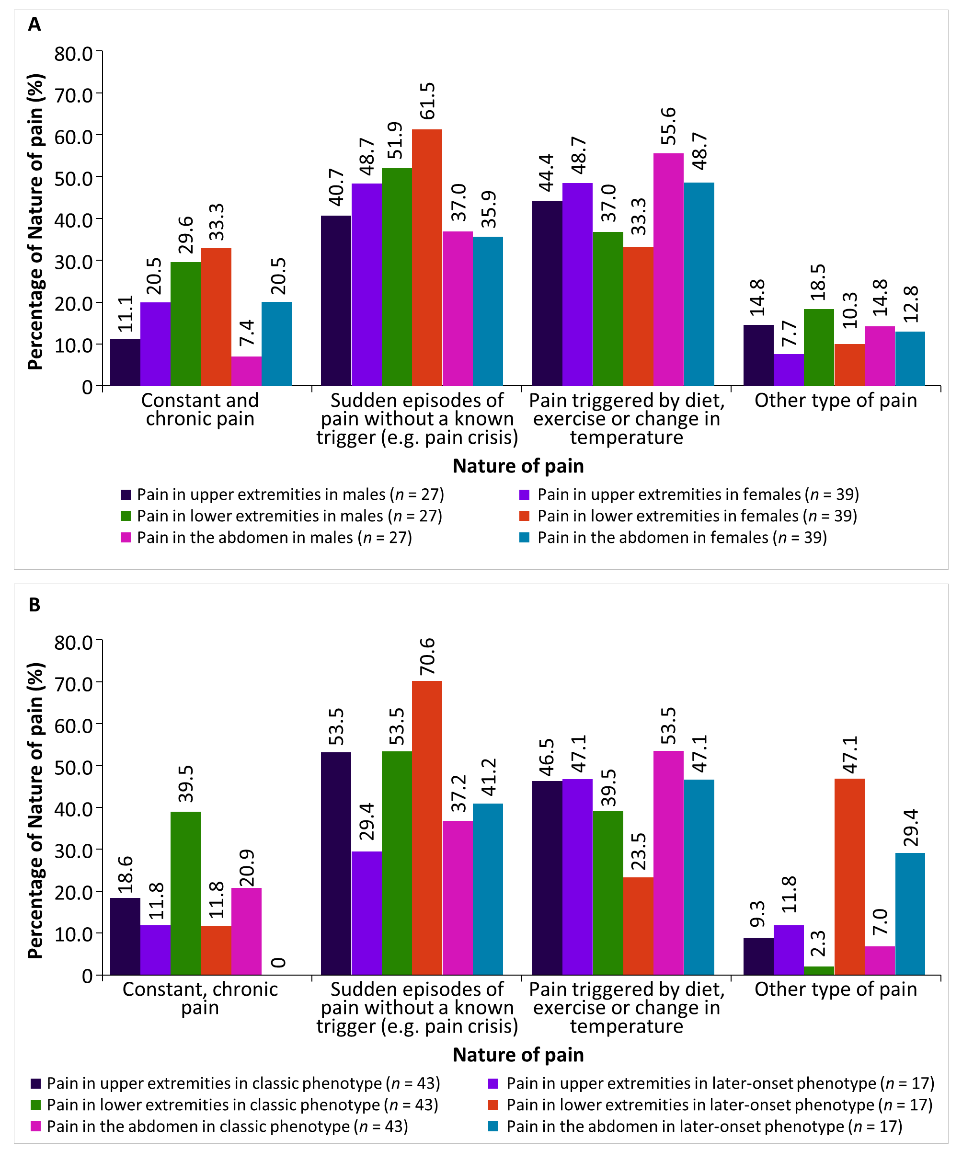


Supplementary Fig. 2 Nature of pain across sex (A) and phenotype (B) by location in participants with FD. FD, Fabry disease; *n*, number of participants


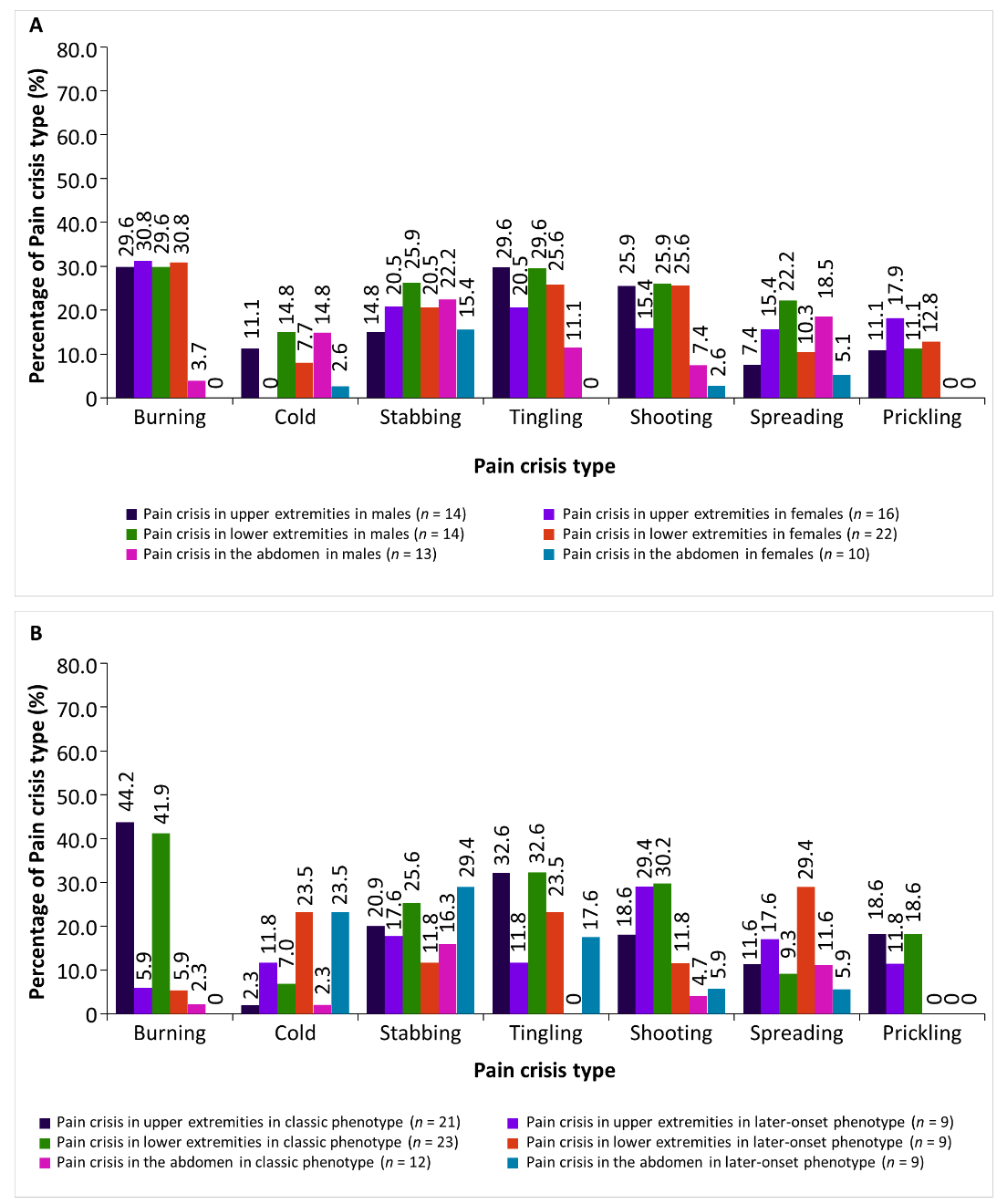


Supplementary Fig. 3 Pain crisis type across sex (A) and phenotype (B) by location in participants with FD. FD, Fabry disease; *n*, number of participants


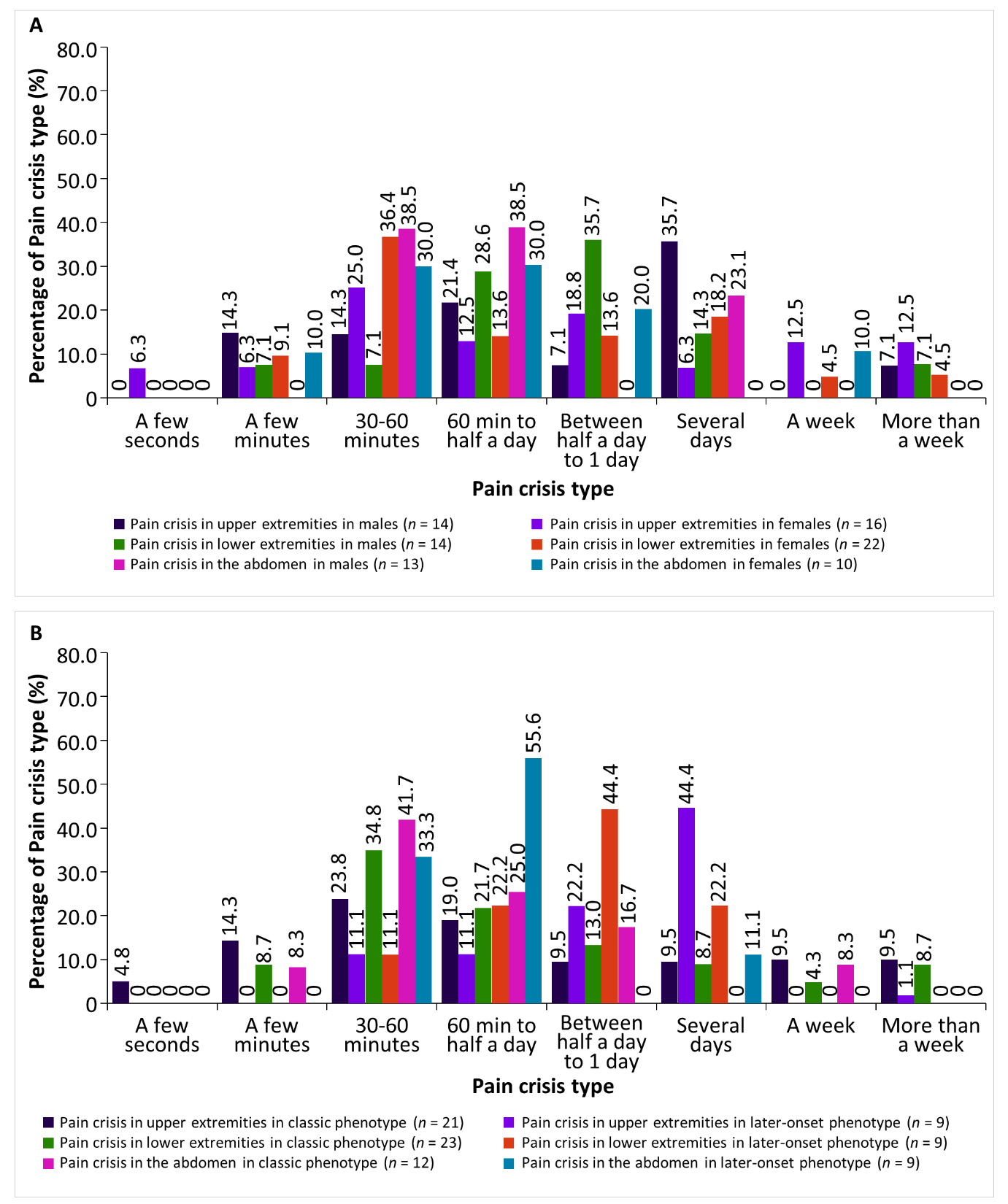


Supplementary Fig. 4 Pain duration profile across sex (A) and phenotype (B) by location in participants with FD. FD, Fabry disease; *n*, number of participants

Supplementary Table 1 FD-PRO symptom severity score

| **FD-PRO symptoms (*N* = 66)** | **Mean (**±**SD)** |
| --- | --- |
| Item 1: Pain in your hands or arms | 5.2 (±2.5) |
| Item 2: Burning in your hands or arms | 4.7 (±2.7) |
| Item 3: Numbness in your hands or arms | 4.3 (±3.0) |
| Item 4: Tingling in your hands or arms | 4.2 (±2.9) |
| Item 5: Pain in your feet or legs | 5.7 (±2.7) |
| Item 6: Burning in your feet or legs | 4.8 (±2.6) |
| Item 7: Numbness in your feet or legs | 4.1 (±3.1) |
| Item 8: Tingling in your feet or legs | 4.4 (±2.9) |
| Item 9: Headache | 4.0 (±3.1) |
| Item 10: Abdominal pain (stomach and/or intestinal) | 4.6 (±2.9) |
| Item 11: Feeling too hot | 5.7 (±2.8) |
| Item 12: Swelling in your legs and feet | 4.1 (±3.2) |
| Item 13: Ringing or buzzing in your ear(s) | 4.0 (±3.2) |
| Item 14: Feeling tired | 6.6 (±2.3) |
| Item 15: Hearing problems | 3.7 (±3.2) |
| Item 16: Vision problems | 3.2 (±3.2) |
| Item 17: Sweating***** | 3.6 (±3.3) |
| Item 18: Difficulty engaging in regular physical activities | 5.5 (±3.0) |

**n* = 45.

FD-PRO, Fabry Disease Patient-Reported Outcome; *n*, number of participants; SD, standard deviation
